# Resting-state EEG data before and after cognitive activity across the adult lifespan and a 5-year follow-up

**DOI:** 10.1038/s41597-024-03797-w

**Published:** 2024-09-10

**Authors:** Stephan Getzmann, Patrick D. Gajewski, Daniel Schneider, Edmund Wascher

**Affiliations:** 1 Leibniz Research Centre for Working Environment and Human Factors at the Technical University of Dortmund (IfADo), Dortmund, Germany; 2 German Center for Mental Health (DZPG), partner site Bochum/Marburg, Bochum, Germany

**Keywords:** Neuroscience, Cognitive ageing, Cognitive neuroscience, Electroencephalography - EEG

## Abstract

This dataset consists of 64-channels resting-state EEG recordings of 608 participants aged between 20 and 70 years, 61.8% female, as well as follow-up measurements after approximately 5 years of 208 participants, starting 2021. The EEG was measured for three minutes with eyes open and eyes closed before and after a 2-hour block of cognitive experimental tasks. The data set is part of the Dortmund Vital Study, a prospective study on the determinants of healthy cognitive aging. The dataset can be used for (1) analyzing cross-sectional resting-state EEG of healthy individuals across the adult life span; (2) generating normalization data sets for comparison of resting-state EEG data of patients with clinically relevant disorders; (3) studying effects of performing cognitive tasks on resting-state EEG and age; (4) exploring intra-individual changes in resting-state EEG and effects of task performance over a time period of about 5 years. The data are provided in Brain Imaging Data Structure (BIDS) format and are available on OpenNeuro.

## Background & Summary

Resting-state EEG (rs-EEG) is a non-invasive measure of the spontaneous electrical activity of the brain, resulting from the synchronized firing of ensembles of neurons in a given frequency (for review^1^). rs-EEG is measured during a state of rest, that is, while remaining still and relaxed, and without performing any assigned cognitive tasks. It therefore provides a window into the intrinsic connectivity and functional organization of the brain, when not actively engaged in a specific task. rs-EEG is traditionally characterized by specific features in the frequency bands of the power spectrum, which are referred to as delta (0.5–4 Hz), theta (4–8 Hz), alpha (8–12 Hz), beta (12–30 Hz), and gamma (>30 Hz) waves^1,2^. Changes in these features have been associated with numerous psychiatric disorders, including depression, attention deficit-hyperactivity disorder, or schizophrenia (for review^3^). For example, dementia such as the Alzheimer’s disease are often linked to typical changes in rs-EEG, such as slowing (i.e., a shift from high-frequency towards low-frequency components) and reduced complexity and synchronization^4^.

However, normal aging is also associated with characteristic dynamics in rs-EEG, based on neurobiological changes of the healthy brain (for review^5^). Studies comparing adolescents and adults showed that EEG power typically decreases with increasing age, while functional networks become more organized, probably due to structural changes of the brain^1^. During adulthood, age-related changes in brain wave frequency, power, morphology, and topography are typically characterized by a reduced amplitude of and slowing in alpha activity. There is also an increase in beta power, while partly contradictory findings were reported in delta and theta power^6,7^. Moreover, links between rs-EEG and cognition have been described, for instance in a study with healthy adults over a wide age range (18–89 years), in which overall higher levels of power in older individuals were associated with less cognitive decline^8^. In addition, age-independent factors such as motivation and fatigue were found. For example, mental fatigue induced by long time cognitive activity was associated with an increased proportion of low-frequency EEG waves and decrease in high-frequency waves^7,9^. To further enhance our understanding of the brain development across the lifespan, the differences between healthy and clinically relevant groups and their relationships with brain activity, more studies on rs-EEG of healthy subjects over a wide age range appear important, however^1^. This applies especially to longitudinal measurements of changes over the entire adult lifespan.

Here, we describe a dataset that contains 64-channel rs-EEG of 608 participants in the age range of 20 and 70 years, measured before and after performance of a 2-h block of cognitive tasks, as well as follow-up measurements after approximately 5 years of 208 participants. The large age range, the relatively even age distribution of the participants, as well as the longitudinal data of some of them offer scientists the opportunity to investigate cross-sectional rs-EEG of healthy individuals across the adult lifespan, aftereffects of cognitive activity and fatigue on rs-EEG as well as intra-individual changes in rs-EEG over an extended time period of about 5 years. Finally, the data set can be used to generate normalization data sets for comparison of rs-EEG data of groups with clinically relevant disorders.

## Methods

### Participants

The EEG measurements were part of the Dortmund Vital Study, an ongoing longitudinal cohort study on the development of cognitive functions over an age range from 20 to 70 years, conducted by the Leibniz Research Centre for Working Environment and Human Factors at the Technical University Dortmund (IfADo) (ClinicalTrials.gov Identifier: NCT05155397). The participants were recruited from local companies, and public institutions, and through advertisements in newspapers and public media. Exclusion criteria were history of severe diseases, namely neurological diseases (such as dementia, Parkinson disease, or stroke); cardiovascular, oncological, and eye diseases; psychiatric and affective disorders; head injuries, head surgery, and head implants; use of psychotropic drugs and neuroleptics; limited physical fitness and mobility. The intake of medication such as blood thinners, hormones, antihypertensives, and cholesterol reducers did not lead to exclusion from the study. The participants reported to be healthy and free of medication that might affect their attention during the experimental sessions. In general, the study population can be considered as representative in terms of age distribution, genetics, cognitive performance parameters, and occupation, whereas there were differences in gender distribution and educational qualifications compared to the general population in Germany^10^. All participants gave their written informed consent before any study protocol was commenced. The study conformed to the Code of Ethics of the World Medical Association (Declaration of Helsinki) and was approved by the local Ethical Committee of the Leibniz Research Centre for Working Environment and Human Factors, Dortmund, Germany (approval number: A93-1 and A93-3 for the follow-up testing).

rs-EEG data from a total of 608 participants (age range 20–70 years, mean 44.1 years, SD 14.5; 376 female, 61.8%, 232 male, 38.2%; 566 right-handed, 93.1%, 40 left-handed, 6.6%, no information on handedness for two subjects) were recorded. This baseline measurement took place between 2016 and 2023 (session 1). Of these participants, 208 (130 female, 62.5%, 78 male, 37.5%; 201 right-handed, 96.6%, 7 left-handed, 3.4%) took also part in a follow-up measurement conducted between 2021 and 2024 (session 2), approximately 5 years after the baseline measurement.

### Task and procedure

**Fig. 1:**
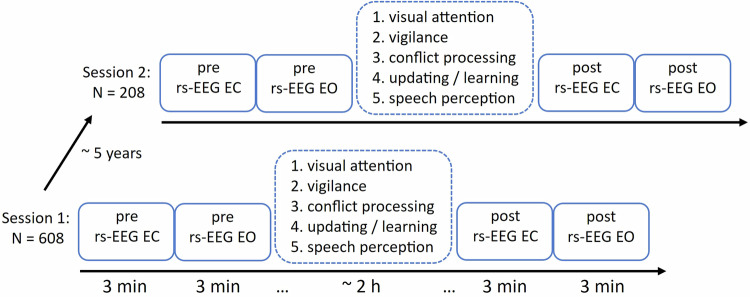
Schematic illustration of the measurement procedure: The resting-state EEG was measured for 3 minutes each with eyes closed (rs-EEG EC) and eyes open (rs-EEG EO) before (pre) and after (post) an approximately 2-hour cognitive test battery, in which five cognitive functions were tested. Of the 608 subjects from the initial measurement (session 1), 208 took part in a follow-up measurement after approximately 5 years (session 2).

The Dortmund Vital Study includes a series of five standardized EEG-based mental tasks in which different cognitive functions are tested^10^ (see also https://vital-study.ifado.de/measures/cognitive-tests-with-eeg-recording/tasks-on-day-1). In brief, visual attention is evaluated by a perceptual control task, in which participants have to respond to luminance changes of one of two symmetrically presented bars^11^; vigilance is tested by a 10-minute psychomotor sustained attention task, in which response times to visual stimuli occurring at random interstimulus intervals are measured^12^; stimulus-response conflict processing is tested by the Simon task, in which spatially arranged responses to nonspatial stimulus features are compared with the task-irrelevant stimulus location and the response are on the same versus opposite sides^13^; updating and statistical learning are evaluated by the AX-Continuous Performance Task, in which participants have to respond to a certain cue-probe pair (i.e., target cue-target probe; AX trials) and to withhold their response or make an alternate response or use other cue-probe pairs^14^; speech-in-noise perception and auditory selective attention are examined in a multi-speaker listening task, in which participants respond to target speech stimuli while ignoring competing speech stimuli^15^. These tasks are carried out one after the other with short breaks, with the entire procedure taking about two hours. In order to promote a state of wakefulness, the testing was conducted during the morning at the same time for all participants. Before the start of the cognitive test battery, the rs-EEG activity is measured for 3 minutes with eyes closed (EC), and 3 minutes with eyes open (EO). The EC condition is always recorded prior to the EO condition. Both methods of rs-EEG measurement differ in a certain way, since EC is suited more as a baseline measure of arousal (reflecting resting-state activity without external stimulation) and EO as a baseline measure of activation (for example, when comparing brain activity at rest and during activity)^16^. In order to assess potential effects of task performance and fatigue on brain activity, the rs-EEG measurements are repeated after the cognitive test battery is completed. Thus, the pre-test and post-test EEG measurements are about two hours apart. The measurement procedure is displayed in Fig. 1. In order to assess changes in rs-EEG and effects of age on a longitudinal basis, all participants who took part in the first measurement (session 1) are invited to a follow-up measurement after approximately 5 years (session 2). The procedure for this ongoing follow-up measurement is exactly the same as for the first measurement.

Recording setup. The rs-EEG was measured using a 64-channel elastic cap arranged based on the 10–20 system with FCz electrode as on-line reference, and a BrainVision Brainamp DC amplifier and BrainVision Recorder software (BrainProducts GmbH). The EEG signal was recorded with a 1000-Hz sampling rate and filtered online by a 250-Hz low-pass filter. No online high-pass filter was used. Impedances were kept below 10 kΩ.

## Data Records

**Fig. 2:**
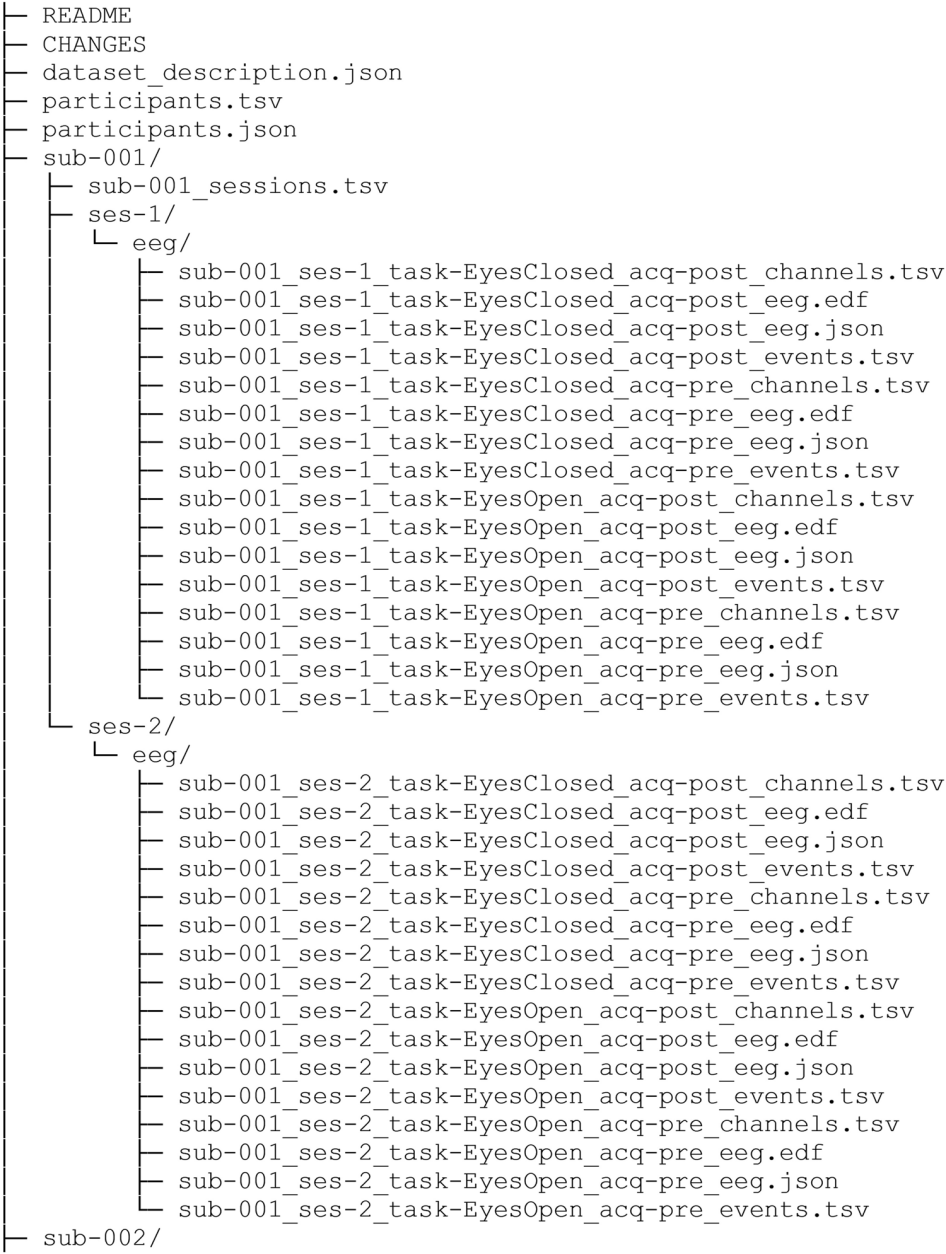
Data structure: For each subject, information on the first and second (if available) measurement time (session 1 and session 2) is specified (_sessions.tsv), and for each session EEG files of four measurement conditions are provided, containing resting-state EEG with eyes closed (EyesClosed) and eyes open (EyesOpen), before (acq-pre) and after (post) the cognitive test battery. For each measurement condition four files are provided, containing channel information (_channel.tsv), EEG measurement information (_eeg.json), trigger information (_event.tsv) and the raw EEG data (_eeg.edf).

The dataset is available at OpenNeuro (dataset accession number: ds005385, 10.18112/openneuro.ds005385.v1.0.2)^17^ in BIDS format^18^. The data structure is shown in Fig. 2. Information about the participants regarding age, sex, handedness, and the availability of data for the first measurement (session 1) and the follow-up measurement (session 2) are provided in the “participants.tsv” file in the main folder. These variables are described in detail in the “participants.json” file. Information on the dataset and general information on the measurement procedure are given in the “dataset_description.json” and “README.md” files. For each subject, subfolders are provided, containing information on the first (session 1) and – if available – second measurement time (session 2) in the “_sessions.tsv” file. Furthermore, there are subfolders for session 1 (“ses-1”) and session 2 (“ses-2”, if available), containing 16 files each. Here, files of four measurement conditions are provided, containing resting state EEG with eyes closed (EyesClosed) and eyes open (EyesOpen), before (acq-pre) and after (post) the cognitive test battery. For each measurement condition four files are given, containing channel information (“_channel.tsv”), EEG measurement information (“_eeg.json”), trigger information (“_event.tsv”) and the raw EEG data (“_eeg.edf”).

## Technical Validation

The data was checked for completeness and includes the non-preprocessed raw EEG.

An estimate of the reliability of the rs-EEG data was provided by a study, in which the intra-class correlation (ICC) in absolute EEG alpha power (8–13 Hz) of all four recordings at the first measurement (session 1) was examined on selected frontal and parietal electrodes in a subgroup of 370 participants^19^. The ICC ranged between 0.92 and 0.94 in the EC condition and between 0.87 and 0.90 in the EO condition, indicating good to excellent ratings of alpha power reliability, depending on interpretation criteria^20,21^. A recent analysis of the reliability of EEG microstate indicated good to excellent short-term retest-reliability of microstate durations, occurrences, and coverages in a subgroup of 583 participants, as well as good overall short, intermediate, and long-term re-test reliability of these microstate characteristics across session 1 and session 2, covering a period of more than half a year^22^.

## Data Availability

n/a.
